# Supplementary material for: SERPINE1 DNA Methylation Levels Quantified in Blood Cells at Five Years of Age Are Associated with Adiposity and Plasma PAI-1 Levels at Five Years of Age
Source: Int J Mol Sci. 2022 Oct 5;23(19):11833. doi: 10.3390/ijms231911833 (PMC9569956; doi:10.3390/ijms231911833)
Supplement: Supplementary file 1 [file ijms-23-11833-s001.zip › ijms-1899725-supplementary.pdf]

Supplementary Table S1. Genomic localization of annotated CpG sites within *SERPINE1* gene locus

**CpGs**	**Genomic Location**
cg20773815	chr7: 100, 768, 975
cg24539923	chr7: 100, 769, 903
cg19722814	chr7: 100, 769, 933
cg25826546	chr7: 100, 770, 061
cg20438404	chr7: 100, 770, 193
cg08506775	chr7: 100, 770, 286
cg02273392	chr7: 100, 770, 415
cg15874872	chr7: 100, 770, 435
cg20583316	chr7: 100, 771, 476
cg01971264	chr7: 100, 773, 079
cg08792542	chr7: 100, 773, 718
cg12584355	chr7: 100, 773, 852
cg11353706	chr7: 100, 774, 811
cg01975495	chr7: 100, 779, 018
cg02704552	chr7: 100, 781, 178

Note : The genomic location of each of the CpG sites was reported using the GRCh37/hg19 version of human genome [26]. Abbreviation: CpGs, Cytosine-phosphate-Guanine.

Supplementary Table S2. Correlation map of DNA methylation levels in blood cells collected at 5 years of age in children (boys and girls) for 15 CpG sites annotated at *SERPINE1* locus.

**Boys and girls**	**20773815**	**24539923**	**19722814**	**25826546**	**20438404**	**08506775**	**02273392**	**15874872**	**20583316**	**01971264**	**08792542**	**12584355**	**11353706**	**01975495**	**02704552**
**n = 323**	0.87	0.06	0.04	0.04	0.03	0.05	0.02	0.04	0.03	0.93	0.95	0.93	0.83	0.30	0.79
**Mean % (SD)**	(0.02)	(0.01)	(0.01)	(0.01)	(0.01)	(0.02)	(0.01)	(0.01)	(0.01)	(0.01)	(0.01)	(0.01)	(0.02)	(0.04)	(0.04)
**cg20773815**	1	0.075	**-0.122**	0.025	-0.040	**-0.196***	-0.017	0.030	-0.016	**0.153***	**0.166***	**0.123**	**0.161***	-0.051	0.064
		0.176	**0.029**	0.654	0.477	**<0.001**	0.761	0.594	0.776	**0.006**	**0.003**	**0.027**	**0.004**	0.361	0.255
**cg24539923**		1	**0.230***	0.053	-0.023	**0.120**	0.056	**0.140**	0.100	**-0.122**	**-0.122**	0.048	0.022	**0.123**	-0.010
			**<0.001**	0.344	0.680	**0.031**	0.312	**0.012**	0.073	**0.029**	**0.028**	0.393	0.689	**0.028**	0.852
**cg19722814**			1	**0.167***	**0.134**	0.027	0.104	0.075	0.107	**-0.273***	**-0.150***	0.092	0.106	**0.256***	0.106
				**0.003**	**0.016**	0.629	0.062	0.178	0.056	**<0.001**	**0.007**	0.099	0.056	**<0.001**	0.058
**cg25826546**				1	**0.145***	0.039	0.047	0.013	**0.110**	**-0.114**	-0.078	-0.053	**0.140**	**0.149***	0.037
					**0.009**	0.489	0.402	0.816	**0.049**	**0.040**	0.160	0.345	**0.012**	**0.007**	0.503
**cg20438404**					1	**-0.152***	**0.129**	0.073	0.050	-0.084	-0.092	0.050	0.092	-0.033	0.028
						**0.006**	**0.021**	0.189	0.370	0.134	0.100	0.374	0.099	0.551	0.618
**cg08506775**						1	0.001	-0.019	-0.018	-0.071	-0.014	-0.050	0.050	0.092	**-0.122**
							0.993	0.729	0.747	0.204	0.803	0.370	0.369	0.098	**0.029**
**cg02273392**							1	**0.151***	0.054	-0.108	0.016	-0.076	0.003	0.014	0.079
								**0.006**	0.333	0.052	0.773	0.174	0.958	0.807	0.155
**cg15874872**								1	-0.054	-0.075	-0.024	0.004	0.016	0.049	-0.041
									0.330	0.178	0.673	0.936	0.773	0.383	0.459
**cg20583316**									1	**-0.162***	-0.058	-0.071	0.021	0.016	-0.076
										**0.003**	0.302	0.204	0.703	0.775	0.171
**cg01971264**										1	**0.257***	0.071	0.014	-0.094	0.081
											**<0.001**	0.203	0.798	0.092	0.146
**cg08792542**											1	0.059	0.065	0.009	0.032
												0.287	0.241	0.869	0.566
**cg12584355**												1	0.058	**0.138**	0.098
													0.298	**0.013**	0.080
**cg11353706**													1	**0.214***	0.028
														**<0.001**	0.619
**cg01975495**														1	**0.244***
															**<0.001**
**cg02704552**															**1**

Note: CpG sites associated with adiposity (P-value < 0.05 before correction for multiple testing) are represented in red and CpG site associated with plasma PAI-1 levels is represented in blue. The first line of each CpG sites corresponds to the Pearson's partial correlation coefficient whereas the second line corresponds to the P-value of the association. Model adjusted for age, sex, batch effect and cell type. Significant results with P-value < 0.05 are in bold. *Significant result with P-value < 0.010.

Supplementary Table S3. Correlation map of DNA methylation levels in blood cells collected at 5 years of age in girls for 15 CpG sites annotated at *SERPINE1* locus.

Girls	20773815	24539923	19722814	25826546	20438404	08506775	02273392	15874872	20583316	01971264	08792542	12584355	11353706	01975495	02704552
n = 146	0.87	0.06	0.04	0.04	0.03	0.06	0.02	0.04	0.03	0.93	0.96	0.93	0.83	0.30	0.80
Mean % (SD)	(0.02)	(0.01)	(0.01)	(0.01)	(0.01)	(0.02)	(0.01)	(0.01)	(0.01)	(0.01)	(0.01)	(0.01)	(0.03)	(0.04)	(0.03)
**cg20773815**	1	-0.006	**-0.226***	-0.037	-0.078	**-0.164**	-0.101	-0.059	-0.078	**0.198**	**0.228***	0.138	**0.249***	-0.085	0.159
		0.939	**0.006**	0.655	0.348	**0.048**	0.227	0.478	0.352	**0.017**	**0.006**	0.096	**0.002**	0.309	0.055
**cg24539923**		1	**0.302***	-0.014	-0.055	**0.267***	0.132	0.103	0.098	**-0.211**	-0.111	0.038	-0.035	0.135	0.014
			**<0.001**	0.867	0.507	**0.001**	0.111	0.218	0.239	**0.010**	0.183	0.646	0.671	0.105	0.866
**cg19722814**			1	**0.259***	0.058	0.039	0.064	0.007	0.146	**-0.295***	**-0.222***	0.072	0.155	**0.316***	**0.187**
				**0.002**	0.487	0.639	0.442	0.930	0.079	**<0.001**	**0.007**	0.389	0.061	**<0.001**	**0.024**
**cg25826546**				1	**0.235***	-0.011	0.077	**0.179**	0.131	**-0.204**	-0.122	-0.113	**0.267***	**0.221***	0.091
					**0.004**	0.895	0.357	**0.030**	0.114	**0.013**	0.143	0.175	**0.001**	**0.007**	0.274
**cg20438404**					1	-0.158	0.022	0.029	0.039	-0.083	-0.148	0.064	**0.201**	-0.016	-0.146
						0.057	0.789	0.731	0.641	0.317	0.075	0.440	**0.015**	0.849	0.079
**cg08506775**						1	-0.025	0.008	-0.034	-0.140	-0.094	-0.058	-0.064	0.060	-0.152
							0.766	0.928	0.687	0.093	0.259	0.484	0.441	0.471	0.067
**cg02273392**							1	**0.196**	0.118	-0.042	0.012	-0.056	0.041	0.111	0.110
								**0.018**	0.158	0.615	0.881	0.500	0.624	0.183	0.186
**cg15874872**								1	-0.104	-0.067	0.026	0.012	-0.075	-0.004	-0.054
									0.210	0.419	0.755	0.883	0.366	0.965	0.518
**cg20583316**									1	**-0.302***	-0.052	-0.008	0.072	0.080	-0.082
										**<0.001**	0.533	0.924	0.388	0.339	0.323
**cg01971264**										1	**0.256***	0.034	-0.041	-0.036	0.027
											**0.002**	0.682	0.621	0.670	0.742
**cg08792542**											1	0.087	0.144	0.006	-0.004
												0.297	0.082	0.940	0.958
**cg12584355**												1	0.034	0.100	-0.001
													0.683	0.228	0.995
**cg11353706**													1	**0.221***	0.015
														**0.007**	0.856
**cg01975495**														1	**0.288***
															**<0.001**
**cg02704552**															1

Note: CpG sites associated with adiposity (P-value < 0.05 before correction for multiple testing) are represented in red and CpG site associated with plasma PAI-1 levels is represented in blue. The first line of each CpG sites corresponds to the Pearson's partial correlation coefficient whereas the second line corresponds to the P-value of the association. Model adjusted for age, batch effect and cell type. Significant results with P-value < 0.05 are in bold. *Significant result with P-value < 0.010.

Supplementary Table S4. Correlation map of DNA methylation levels in blood cells collected at 5 years of age in boys for 15 CpG sites annotated at *SERPINE1* locus.

**Boys**	**20773815**	**24539923**	**19722814**	**25826546**	**20438404**	**08506775**	**02273392**	**15874872**	**20583316**	**01971264**	**08792542**	**12584355**	**11353706**	**01975495**	**02704552**
**n = 177**	0.87	0.06	0.04	0.04	0.03	0.05	0.02	0.04	0.03	0.93	0.95	0.93	0.83	0.30	0.79
**Mean % (SD)**	(0.01)	(0.01)	(0.01)	(0.01)	(0.01)	(0.02)	(0.01)	(0.01)	(0.01)	(0.01)	(0.01)	(0.01)	(0.02)	(0.05)	(0.04)
**cg20773815**	1	**0.162**	-0.011	0.102	-0.004	**-0.223***	0.055	0.117	0.041	0.113	0.106	0.111	0.075	-0.022	-0.008
		**0.031**	0.881	0.175	0.956	**0.003**	0.471	0.120	0.592	0.133	0.159	0.142	0.319	0.769	0.920
**cg24539923**		1	**0.172**	0.105	0.008	-0.007	0.004	**0.177**	0.103	-0.050	-0.143	0.047	0.070	0.112	-0.042
			**0.022**	0.164	0.917	0.931	0.959	**0.019**	0.174	0.512	0.058	0.533	0.357	0.137	0.580
**cg19722814**			1	0.083	**0.208***	0.036	0.133	0.143	0.070	**-0.251***	-0.066	0.126	0.064	**0.214***	0.055
				0.274	**0.006**	0.634	0.077	0.058	0.352	**0.001**	0.382	0.096	0.400	**0.004**	0.465
**cg25826546**				1	0.063	0.057	0.034	**-0.155**	0.092	-0.036	-0.047	-0.003	-0.001	0.087	-0.026
					0.407	0.449	0.650	**0.039**	0.223	0.638	0.535	0.967	0.988	0.249	0.728
**cg20438404**					1	-0.145	**0.211***	0.112	0.059	-0.083	-0.040	0.039	-0.003	-0.046	**0.155**
						0.054	**0.005**	0.139	0.435	0.273	0.599	0.610	0.970	0.546	**0.039**
**cg08506775**						1	0.032	-0.037	-0.007	-0.028	0.036	-0.059	0.131	0.111	-0.124
							0.676	0.624	0.931	0.707	0.632	0.435	0.083	0.143	0.101
**cg02273392**							1	0.114	0.006	**-0.157**	0.026	-0.086	-0.024	-0.052	0.070
								0.131	0.942	**0.037**	0.729	0.253	0.755	0.488	0.356
**cg15874872**								1	-0.012	-0.081	-0.069	-0.001	0.102	0.090	-0.030
									0.869	0.283	0.364	0.987	0.177	0.233	0.690
**cg20583316**									1	-0.052	-0.063	-0.127	-0.022	-0.030	-0.073
										0.489	0.408	0.091	0.774	0.694	0.337
**cg01971264**										1	**0.256***	0.101	0.060	-0.137	0.115
											**0.001**	0.179	0.425	0.068	0.127
**cg08792542**											1	0.025	-0.014	0.010	0.051
												0.739	0.850	0.897	0.498
**cg12584355**												1	0.076	**0.169**	**0.167**
													0.312	**0.025**	**0.027**
**cg11353706**													1	**0.209***	0.031
														**0.005**	0.685
**cg01975495**														1	**0.216***
															**0.004**
**cg02704552**															1

Note: CpG sites associated with adiposity (P-value < 0.05 before correction for multiple testing) are represented in red and CpG site associated with plasma PAI-1 levels is represented in blue. The first line of each CpG sites corresponds to the Pearson's partial correlation coefficient whereas the second line corresponds to the P-value of the association. Model adjusted for age, batch effect and cell type. Significant results with P-value < 0.05 are in bold. *Significant result with P-value < 0.010.

Supplementary Table S5. Associations between plasma PAI-1 levels and adiposity markers in all children aged 5 years old with values of an interaction term between plasma PAI-1 levels and sex for each of the associations tested.

	**Log_{10} Waist circumference[a], cm N = 341**	**Log_{10} DXA trunk fat[a], % N = 308**	**Log_{10} DXA android fat[a], % N = 308**	**Log_{10} DXA total fat, [a] % N = 308**	**Log_{10} Total skinfold[ac], mm N = 291**	**BMI z-score[a] N = 341**
**Log_{10} PAI-1 plasma level, ng/ml**	r = 0.095<br>p = 0.081	r = 0.108<br>p = 0.059	r = 0.092<br>p = 0.109	r = 0.104<br>p = 0.070	r = 0.058<br>p = 0.287	r = 0.036<br>p = 0.506
Interaction term						
**Log_{10} PAI-1 plasma level, ng/ml**	β_{PAI-1}: 0.014<br>β_{inter}: -0.008<br>p_{inter}: 0.477	β_{PAI-1}: 0.018<br>β_{inter}: 0.008<br>p_{inter}: 0.725	β_{PAI-1}: 0.018<br>β_{inter}: 0.005<br>p_{inter}: 0.838	β_{PAI-1}: 0.014<br>β_{inter}: 0.008<br>p_{inter}: 0.675	β_{PAI-1}: 0.036<br>β_{inter}: -0.026<br>p_{inter}: 0.549	β_{PAI-1}: 0.218<br>β_{inter}: -0.198<br>p_{inter}: 0.570

Note: r is the Pearson's partial correlation coefficient. β_{PAI-1}: Regression coefficient of plasma PAI-1 levels in girls. β_{inter}: Regression coefficient of the interaction term (plasma PAI-1*sex). P_{inter}: P-value of the interaction term. [a]Model adjusted for age, sex, batch effect and cell type heterogeneity. Abbreviations: BMI, body mass index; DXA, Dual-energy X-ray absorptiometry. [c]Total skinfolds thickness is the sum of biceps, triceps, subscapular and suprailiac skinfolds.

Supplementary Table S6. Associations between blood cells DNAm and adiposity markers and plasma PAI-1 levels in all children aged 5 years old and stratified by sex only for CpG sites with an interaction term.

	**Adiposity markers**						**Log10 PAI-1 plasma level, ng/ml[b]**
	**Log10 Waist circumference[a], cm**	**Log10 DXA trunk fat[a], %**	**Log10 DXA android fat[a], %**	**Log10 DXA total fat [a], %**	**Log10 Total skinfold[ac], mm**	**BMI z-score[a]**	
			**All children**				
	N = 323	N = 297	N = 297	N = 297	N = 322	N = 323	N = 321
**cg20773815**	r = -0.070<br>p = 0.219	r = -0.051<br>p = 0.392	r = -0.089<br>p = 0.131	r = -0.044<br>p = 0.461	r = -0.117<br>p = 0.038	r = -0.059<br>p = 0.297	r = 0.041<br>p = 0.469
**cg24539923**	r = -0.032<br>p = 0.566	r = 0.004<br>p = 0.948	r = 0.008<br>p = 0896	r = 0.023<br>p = 0.696	r = 0.011<br>p = 0.852	r = 0.031<br>p = 0.582	r = -0.074<br>p = 0.194
**cg19722814**	r = -0.072<br>p = 0.203	r = -0.076<br>p = 0.197	r = -0.057<br>p = 0.338	r = -0.049<br>p = 0.406	r = -0.052<br>p = 0.362	r = -0.064<br>p = 0.262	r = -0.097<br>p = 0.088
**cg25826546**	r = -0.006<br>p = 0.921	r = -0.096<br>p = 0.103	r = -0.088<br>p = 0.135	r = -0.102<br>p = 0.085	r = -0.035<br>p = 0.537	r = -0.052<br>p = 0.360	r = -0.076<br>p = 0.179
**cg20438404**	r = -0.013<br>p = 0.824	r = -0.029<br>p = 0.624	r = -0.017<br>p = 0.770	r = -0.053<br>p = 0.371	r = -0034<br>p = 0.554	r = -0.028<br>p = 0.617	r = 0.075<br>p = 0.183
**cg08506775**	r = 0.055<br>p = 0.333	r = 0.033<br>p = 0.582	r = 0.084<br>p = 0.153	r = 0.070<br>p = 0.236	r = 0.096<br>p = 0.089	r = 0.083<br>p = 0.140	r = 0.064<br>p = 0.261
**cg02273392**	r = -0.042<br>p = 0.463	r = -0.019<br>p = 0.754	r = -0.006<br>p = 0.914	r = 0.001<br>p = 0.991	r = -0.054<br>p = 0.338	r = -0.036<br>p = 0.520	r = -0.035<br>p = 0.538
**cg15874872**	r = -0.031<br>p = 0.589	r = -0.007<br>p = 0.908	r = 0.028<br>p = 0.634	r = 0.011<br>p = 0.849	r = -0.046<br>p = 0.415	r = 0.001<br>p = 0.981	r = 0.086<br>p = 0.129
**cg20583316**	r = 0.015<br>p = 0.796	r = 0.053<br>p = 0.371	r = 0.063<br>p = 0.285	r = 0.055<br>p = 0.348	r = 0.064<br>p = 0.256	r = 0.031<br>p = 0.590	r = 0.026<br>p = 0.642
**cg01971264**	r = -0.001<br>p = 0.992	r = 0.124<br>p = 0.035	r = 0.082<br>p = 0.167	r = 0.094<br>p = 0.110	r = 0.071<br>p = 0.207	r = 0.036<br>p = 0.525	r = 0.062<br>p = 0.275
**cg08792542**	r = -0.022<br>p = 0.699	r = -0.001<br>p = 0.982	r = -0.017<br>p = 0.768	r = 0.000<br>p = 0.997	r = -0.016<br>p = 0.780	r = -0.016<br>p = 0.782	r = 0.072<br>p = 0.202
**cg12584355**	r = 0.049<br>p = 0.387	r = -0.017<br>p = 0.777	r = 0.066<br>p = 0.263	r = -0.054<br>p = 0.357	r = -0.031<br>p = 0.587	r = -0.040<br>p = 0.475	r = -0.081<br>p = 0.155
**cg11353706**	r = -0.127<br>p = 0.024	r = 0.777<br>p = -0.126	r = -0.130<br>p = 0.028	r = -0.107<br>p = 0.070	r = -0.085<br>p = 0.136	r = -0.085<br>p = 0.133	r = 0.128<br>p = 0.023
**cg01975495**	r = -0.072<br>p = 0.203	r = -0.030<br>p = 0.610	r = -0.013<br>p = 0.823	r = -0.048<br>p = 0.420	r = -0.049<br>p = 0.384	r = -0.053<br>p = 0.352	r = 0.053<br>p = 0.352
**cg02704552**	r = 0.053<br>p = 0.349	r = 0.610<br>p = 0.047	r = 0.024<br>p = 0.690	r = 0.041<br>p = 0.488	r = 0.020<br>p = 0.727	r = 0.063<br>p = 0.267	r = -0.083<br>p = 0.145
			**Girls**				
	N = 146	N = 136	N = 136	N = 136	N = 145	N = 146	N = 144
**cg25826546**	r = -0.054<br>P = 0.532	r = -0.192<br>p = 0.030	r = -0.151<br>P = 0.089	r = -0.207<br>p = 0.019	r = -0.032<br>P = 0.711	r = -0.084<br>p = 0.326	
**cg11353706**	**r = -0.258**<br>**p = 0.002**	r = -0.189<br>p = 0.032	**r = -0.215**<br>**p = 0.015**	r = -0.203<br>p = 0.021	**r = -0.212**<br>**p = 0.013**	**r = -0.278**<br>**p < 0.001**	
**cg01975495**	r = -0.174<br>p = 0.042	r = -0.152<br>p = 0.086	r = -0.154<br>p = 0.082	r = -0.207<br>p = 0.019	r = -0.152<br>p = 0.076	r = -0.146<br>p = 0.088	
**cg02273392**	r = 0.109<br>p = 0.220	r = 0.134<br>p = 0.133	r = 0.151<br>p = 0.090	r = 0.172<br>p = 0.052	r = 0.150<br>p = 0.090	r = 0.111<br>p = 0.212	
**cg19722814**							r = -0.056<br>p = 0.515
			**Boys**				
	N = 177	N = 161	N = 161	N = 161	N = 177	N = 177	N = 177
**cg25826546**	r = 0.097<br>p = 0.233	r = 0.043<br>p = 0.594	r = 0.016<br>p = 0.843	r = 0.044<br>p = 0.593	r = 0.021<br>p = 0.796	r = 0.002<br>p = 0.977	
**cg11353706**	r = 0.024	r = 0.018	r = 0.034	r = 0.035	r = 0.050	r = 0.095	

	p = 0.767	p = 0.829	p = 0.673	p = 0.667	p = 0.537	p = 0.243	
**cg01975495**	r = 0.038<br>p = 0.645	r = 0.081<br>0.319	r = 0.125<br>p = 0.124	r = 0.090<br>p = 0.269	r = 0.022<br>p = 0.789	r = 0.019<br>p = 0.817	
**cg02273392**	r = -0.155<br>p = 0.041	r = -0.148<br>p = 0.063	r = -0.146<br>p = 0.067	r = -0.130<br>p = 0.102	r = -0.172<br>p = 0.023	r = -0.112<br>p = 0.139	
**cg19722814**							**r = -0.178**<br>**p = 0.021**

Note: r is the Pearson's partial correlation coefficient. [a]Model adjusted for age, sex (only when tested in all children together), batch effect and cell type heterogeneity and significant result with P-value <0.017 are in bold (considering three independent groups of adiposity markers). [b]Model additionally adjusted for BMI and significant P-value < 0.050 are in bold. Abbreviations: BMI, body mass index; DXA, Dual-energy X-ray absorptiometry. [c]Total skinfolds thickness is the sum of biceps, triceps, subscapular and suprailiac skinfolds.

Supplementary Table S7. Interaction term between DNA methylation and sex and its association with adiposity markers and plasma PAI-1 levels.

DNAm level (M-value)	Adiposity markers						Log_{10} PAI-1, pg/ml
	Log_{10} Waist circumference, cm	Log_{10} DXA trunk fat, %	Log_{10} DXA android fat, %	Log_{10} DXA total fat, %	Log_{10} Total skinfold[a], mm	BMI z-score	
**cg20773815**	β_{met}: -0.017<br>β_{inter}: 0.011<br>p_{inter}: 0.541	β_{met}: -0.028<br>β_{inter}: 0.026<br>p_{inter}: 0.482	β_{met}: -0.055<br>β_{inter}: 0.0.53<br>p_{inter}: 0.202	β_{met}: -0.014<br>β_{inter}: 0.006<br>p_{inter}: 0.864	β_{met}: -0.111<br>β_{inter}: 0.082<br>p_{inter}: 0.246	β_{met}: -0.374<br>β_{inter}: 0.175<br>p_{inter}: 0.758	β_{met}: 0.103<br>β_{inter}: -0.083<br>p_{inter}: 0.643
**cg24539923**	β_{met}: 0.002<br>β_{inter}: -0.009<br>p_{inter}: 0.379	β_{met}: 0.011<br>β_{inter}: -0.022<br>p_{inter}: 0.314	β_{met}: 0.003<br>β_{inter}: -0.002<br>p_{inter}: 0.928	β_{met}: 0.014<br>β_{inter}: -0.021<br>p_{inter}: 0.282	β_{met}: 0.042<br>β_{inter}: -0.075<br>p_{inter}: 0.066	β_{met}: 0.258<br>β_{inter}: -0.322<br>p_{inter}: 0.322	β_{met}: -0.040<br>β_{inter}: -0.055<br>p_{inter}: 0.592
**cg19722814**	β_{met}: -0.007<br>β_{inter}: 0.001<br>p_{inter}: 0.869	β_{met}: -0.020<br>β_{inter}: 0.015<br>p_{inter}: 0.361	β_{met}: -0.021<br>β_{inter}: 0.022<br>p_{inter}: 0.223	β_{met}: -0.015<br>β_{inter}: 0.016<br>p_{inter}: 0.255	β_{met}: -0.024<br>β_{inter}: 0.015<br>p_{inter}: 0.614	β_{met}: -0.253<br>β_{inter}: 0.181<br>p_{inter}: 0.456	**β_{met}: 0.003**<br>**β_{inter}: -0.180**<br>**p_{inter}: 0.018**
**cg25826546**	β_{met}: -0.009<br>β_{inter}: 0.018<br>p_{inter}: 0.098	**β_{met}: -0.046**<br>**β_{inter}: 0.062**<br>**p_{inter}: 0.005**	**β_{met}: -0.043**<br>**β_{inter}: 0.054**<br>**p_{inter}: 0.025**	**β_{met}: -0.040**<br>**β_{inter}: 0.053**<br>**p_{inter}: 0.006**	β_{met}: -0.030<br>β_{inter}: 0.038<br>p_{inter}: 0.356	β_{met}: -0.323<br>β_{inter}: 0.374<br>p_{inter}: 0.259	β_{met}: -0.088<br>β_{inter}: 0.038<br>p_{inter}: 0.718
**cg20438404**	β_{met}: 0.001<br>β_{inter}: -0.004<br>p_{inter}: 0.740	β_{met}: -0.006<br>β_{inter}: 0.001<br>p_{inter}: 0.979	β_{met}: 0.004<br>β_{inter}: -0.015<br>p_{inter}: 0.521	β_{met}: -0.013<br>β_{inter}: 0.008<br>p_{inter}: 0.677	β_{met}: -0.007<br>β_{inter}: -0.009<br>p_{inter}: 0.816	β_{met}: -0.170<br>β_{inter}: 0.165<br>p_{inter}: 0.613	β_{met}: 0.138<br>β_{inter}: -0.129<br>p_{inter}: 0.206
**cg08506775**	β_{met}: 0.004<br>β_{inter}: -0.003<br>p_{inter}: 0.320	β_{met}: 0.004<br>β_{inter}: -0.004<br>p_{inter}: 0.528	β_{met}: 0.007<br>β_{inter}: -0.002<br>p_{inter}: 0.734	β_{met}: 0.006<br>β_{inter}: -0.004<br>p_{inter}: 0.442	β_{met}: 0.020<br>β_{inter}: -0.016<br>p_{inter}: 0.188	β_{met}: 0.149<br>β_{inter}: -0.127<br>p_{inter}: 0.197	β_{met}: 0.002<br>β_{inter}: 0.025<br>p_{inter}: 0.432
**cg02273392**	β_{met}: 0.005<br>β_{inter}: -0.015<br>p_{inter}: 0.085	**β_{met}: 0.018**<br>**β_{inter}: -0.037**<br>**p_{inter}: 0.035**	β_{met}: 0.018<br>β_{inter}: -0.034<br>p_{inter}: 0.076	**β_{met}: 0.020**<br>**β_{inter}: -0.035**<br>**p_{inter}: 0.021**	**β_{met}: 0.025**<br>**β_{inter}: -0.071**<br>**p_{inter}: 0.028**	β_{met}: 0.135<br>β_{inter}: -0.382<br>p_{inter}: 0.144	β_{met}: -0.022<br>β_{inter}: -0.006<br>p_{inter}: 0.939
**cg15874872**	β_{met}: -0.004<br>β_{inter}: 0.001<br>p_{inter}: 0.883	β_{met}: -0.011<br>β_{inter}: 0.018<br>p_{inter}: 0.382	β_{met}: 0.000<br>β_{inter}: 0.011<br>p_{inter}: 0.633	β_{met}: -0.006<br>β_{inter}: 0.014<br>p_{inter}: 0.426	β_{met}: -0.015<br>β_{inter}: -0.002<br>p_{inter}: 0.962	β_{met}: -0.151<br>β_{inter}: 0.298<br>p_{inter}: 0.335	β_{met}: 0.142<br>β_{inter}: -0.131<br>p_{inter}: 0.180
**cg20583316**	β_{met}: 0.008<br>β_{inter}: -0.012<br>p_{inter}: 0.292	β_{met}: 0.014<br>β_{inter}: -0.005<br>p_{inter}: 0.823	β_{met}: 0.013<br>β_{inter}: -0.005<br>p_{inter}: 0.973	β_{met}: 0.002<br>β_{inter}: 0.001<br>p_{inter}: 0.545	β_{met}: 0.029<br>β_{inter}: -0.007<br>p_{inter}: 0.875	β_{met}: 0.154<br>β_{inter}: -0.106<br>p_{inter}: 0.764	β_{met}: -0.003<br>β_{inter}: 0.052<br>p_{inter}: 0.638
**cg01971264**	β_{met}: 0.007<br>β_{inter}: -0.012<br>p_{inter}: 0.402	β_{met}: 0.050<br>β_{inter}: -0.031<br>p_{inter}: 0.299	β_{met}: 0.044<br>β_{inter}: -0.036<br>p_{inter}: 0.280	β_{met}: 0.046<br>β_{inter}: -0.044<br>p_{inter}: 0.094	β_{met}: 0.057<br>β_{inter}: -0.036<br>p_{inter}: 0.520	β_{met}: 0.502<br>β_{inter}: -0.641<br>p_{inter}: 0.154	β_{met}: -0.035<br>β_{inter}: 0.209<br>p_{inter}: 0.142
**cg08792542**	β_{met}: -0.018<br>β_{inter}: 0.030<br>p_{inter}: 0.067	β_{met}: -0.006<br>β_{inter}: 0.010<br>p_{inter}: 0.747	β_{met}: -0.002<br>β_{inter}: -0.006<br>p_{inter}: 0.864	β_{met}: 0.006<br>β_{inter}: -0.012<br>p_{inter}: 0.673	β_{met}: -0.051<br>β_{inter}: 0.083<br>p_{inter}: 0.177	β_{met}: -0.376<br>β_{inter}: 0.596<br>p_{inter}: 0.225	β_{met}: 0.034<br>β_{inter}: 0.130<br>p_{inter}: 0.401
**cg12584355**	β_{met}: 0.004<br>β_{inter}: 0.008<br>p_{inter}: 0.681	β_{met}: -0.021<br>β_{inter}: 0.031<br>p_{inter}: 0.435	β_{met}: -0.008<br>β_{inter}: 0.033<br>p_{inter}: 0.452	β_{met}: -0.012<br>β_{inter}: -0.008<br>p_{inter}: 0.815	β_{met}: -0.035<br>β_{inter}: 0.030<br>p_{inter}: 0.680	β_{met}: -0.245<br>β_{inter}: 0.079<br>p_{inter}: 0.891	β_{met}: -0.136<br>β_{inter}: 0.012<br>p_{inter}: 0.946
**cg11353706**	**β_{met}: -0.040**<br>**β_{inter}: 0.044**<br>**p_{inter}: 0.001**	**β_{met}: -0.065**<br>**β_{inter}: 0.066**<br>**p_{inter}: 0.020**	**β_{met}: -0.078**<br>**β_{inter}: 0.081**<br>**p_{inter}: 0.009**	**β_{met}: -0.054**<br>**β_{inter}: 0.059**<br>**p_{inter}: 0.018**	**β_{met}: -0.121**<br>**β_{inter}: 0.153**<br>**p_{inter}: 0.004**	**β_{met}: -1.153**<br>**β_{inter}: 1.561**<br>**p_{inter}: <0.001**	β_{met}: 0.136<br>β_{inter}: 0.043<br>p_{inter}: 0.752
**cg01975495**	**β_{met}: -0.031**<br>**β_{inter}: 0.036**<br>**p_{inter}: 0.002**	**β_{met}: -0.060**<br>**β_{inter}: 0.085**<br>**p_{inter}: <0.001**	**β_{met}: -0.068**<br>**β_{inter}: 0.104**<br>**p_{inter}: <0.001**	**β_{met}: -0.056**<br>**β_{inter}: 0.074**<br>**p_{inter}: <0.001**	**β_{met}: -0.108**<br>**β_{inter}: 0.141**<br>**p_{inter}: 0.001**	**β_{met}: -0.739**<br>**β_{inter}: 0.900**<br>**p_{inter}: 0.011**	β_{met}: 0.035<br>β_{inter}: 0.043<br>p_{inter}: 0.706

**cg02704552**	β_{met}: 0.008<br>β_{inter}: -0.003<br>p_{inter}: 0.819	β_{met}: -0.006<br>β_{inter}: 0.027<br>p_{inter}: 0.276	β_{met}: -0.020<br>β_{inter}: 0.042<br>p_{inter}: 0.128	β_{met}: 0.001<br>β_{inter}: 0.011<br>p_{inter}: 0.614	β_{met}: -0.018<br>β_{inter}: 0.042<br>p_{inter}: 0.357	β_{met}: 0.161<br>β_{inter}: 0.077<br>p_{inter}: 0.833	β_{met}: -0.133<br>β_{inter}: 0.075<br>p_{inter}: 0.513

Note: β_{met}: Regression coefficient of DNAm in girls. β_{inter}: Regression coefficient of the interaction term (DNAm*sex). P_{inter}: P-value of the interaction term. For adiposity markers, regression models are adjusted for age, sex, batch effect and cell type heterogeneity. For PAI-1 plasma levels, models are additionally adjusted for BMI z-score. Significant results with P-value < 0.05 are in bold. [a]Total skinfolds is the sum of biceps, triceps, subscapular and suprailiac skinfolds thickness. Abbreviations: BMI, body mass index; DXA, Dual-energy X-ray absorptiometry.
